# Social determinants of male health: a case study of Leeds, UK

**DOI:** 10.1186/s12889-018-5076-7

**Published:** 2018-01-19

**Authors:** Alan White, Amanda Seims, Ian Cameron, Tim Taylor

**Affiliations:** 1 Centre for Men’s Health, Institute of Health and Wellbeing, Leeds Beckett University, Leeds, LS1 3HE UK; 2 Leeds City Council, Civic Hall, Calverley Street, Leeds, LS1 1UR UK

**Keywords:** Men’s health, Social determinants, Education, Housing, Employment, Relationships

## Abstract

**Background:**

The social determinants of health have a disproportionate impact on mortality in men. A study into the state of health of the male population in Leeds was undertaken to guide public health commissioning decisions. This paper reports on the data relating to the social lives of men.

**Methods:**

A cross-sectional study was undertaken, comprising descriptive analysis of data relating to educational attainment, housing, employment (including benefit claimants), marital status and relationships. Data was considered for the whole city and localised at the Middle Super Output Area (MSOA) level and mapped against the Index of Deprivation.

**Results:**

Boys’ educational attainment was found to be lagging behind girls’ from their earliest assessments (Early Years Foundation Stage Profile, 46% vs. 60%, *P* = 0.00) to GCSEs (53% vs. 63%, *P* = 0.00), leaving many men with no qualifications. There were 68% more men than women identified as being unemployed, with more men claiming benefits. Men living in social housing are more likely to be housed in high-rise flats. Almost 50% of men aged 16–64 are single, with 2254 lone fathers.

**Conclusions:**

There appears to be a lack of sex/gender analysis of current cross city data. In areas of deprivation a complex picture of multiple social problems emerges, with marked gender differences in the social determinants of health, with males seeming to be more negatively affected. There is a need for more focused planning for reaching out and targeting boys and men in the most deprived inner city areas, so that greater efficiency in service delivery can be obtained.

## Background

There is a growing recognition that the health of the male population is heavily affected by their social situation, with increasing poverty linked to a widening gap in life expectancy [1, 2]. This is a global phenomenon, with such differences reported across the European Union [3], Asia [4], Australia [5], and within countries such as Ireland [6], Denmark [7], and Germany [8]. This study explores the social situation of men within a substantial metropolitan city in the UK, however the findings should have a resonance with men in other large conurbations.

The opportunities for improving public health through local action has been recognised [9, 10] and with an increasing agenda for targeting activity within the most challenging areas. Leeds is the third largest city in the United Kingdom; since 2010, there has been a 47% reduction in the money that Leeds City Council gets from Government to run local services. To ensure tendered contracts are fit for purpose, Leeds Council’s Joint Strategic Needs Assessment [11] noted that there was a dearth of information on the 400,000 male population across the city. This acted as a stimulant to instigate a study into the State of Men’s Health in Leeds, to act as a guide to future public health planning.

The findings of that study [12–14] highlighted that there were large differences in both morbidity and mortality for men across different areas of Leeds, suggesting that the health challenges the men were facing were more than a biological issue specific to being male.

There is strong evidence across a wide range of studies and reports on the impact of the wider determinants of health on a population’s wellbeing [15–21]. What has been less well-researched is how being sex is affected by these social factors [22–30]. There has been a sustained campaign for the disaggregation of data by sex in both research and routinely collected data within public health [3, 31–35], with the recent report on women’s health in Europe calling for improved availability and use of sex-disaggregated data that can be cross-linked to social factors [36]. With the push for gender mainstreaming [37] and also the legal requirements for gender to be recognised as a protected group within health policy strategy and service provision [38] the need for such scrutiny is further supported.

Through the more detailed examination of how sex maps onto intersectional factors (such as age, ethnicity, disabilities and sexuality) and the wider social determinants of health a more efficient targeting of resources may be achieved. By making more explicit how social and environmental factors differentially impact on both men and women we can help guide the development of more gender sensitive policies and practices to ensure equity in provision of services as opposed to the blunter push for equality.

The aim of this paper is to provide an overview of the findings from the Leeds study to highlight the wide disparities that exist within our municipalities with regard to some of the key social determinants of men’s health: educational attainment, housing, employment, poverty and living arrangements.

## Methods

A cross-sectional study was adopted, which was based on an examination of routinely collected and available data relating to the social determinants of health.

### Data sources

Educational attainment included the proportion of girls and boys in Leeds achieving: 1) a good level of development in the Early Years Foundation Stage Profile (EYFSP) [39]; 2) level 2 key stage 1 reading; 3) level 2 key stage 1 writing [40]; and 4) three or more higher grade General Certificate of Secondary Education (GCSE) passes including English and Maths [41]. Educational attainment data relating to looked after children was obtained directly from Leeds Children’s Services. Demographic data for those living in Council-owned high-rise flats was obtained directly from the Environments and Housing department within Leeds City Council (obtained April 2015). Benefits data for job seekers allowance and employment and support allowance were obtained from Leeds City Council Public Health intelligence team. Data showing underlying conditions for Employment and Support Allowance (ESA) claimants were obtained from nomisweb.co.uk. Data on divorces were obtained from the Office for National Statistics (ONS) [42].

Census data were used for the following:

- The number of men and women in Leeds with no qualifications [43].
- The number of men and women living alone [44]
- Tenure [45]
- Homelessness [46]
- Unemployment [47]
- Long-term unemployment and ‘never worked’ [48]
- Employment data including status and hours worked [47]
- The number of men and women economically inactive due to a long-term health problem or disability [49]
- Marital status [50]
- Lone fathers [51]

### Data analysis

A descriptive analysis was undertaken. Where possible, data were disaggregated by age group, and sex at a city level and by area of residence within Leeds [at the level of the Middle Super Output Area (MSOA)]. Data were calculated as a proportion of the male or female age-specific population in Leeds or within an MSOA where appropriate. Data for each variable were ranked by MSOA with the greatest occurrence within the male population of that area. Mean ± SD sex differences in prevalence of socio-economic factor were analysed using an independent t-test or Mann Whitney U-test where appropriate, with significance set at *p* < 0.05. Analysis and presentation of data was completed using Microsoft Excel 2013 and SPSS (version 22).

## Results

### Educational attainment

The proportion of boys in Leeds (of all those eligible to be assessed) in 2013 achieving a good level of development in the Early Years Foundation Stage Profile [EYFSP] was significantly lower than for girls (45.6% ± 13.4% as compared to 60.2% ± 13.0%, *P* = 0.00) (Table 1). For both sexes, this was below the national average (boys 52.0%, girls 69.0%). In 3 MSOAs in Leeds the proportion of boys achieving a good level of development was at, or below, 20%.

**Table 1:** Percentage of males and females, for Leeds and national, for Education, housing, employment and marital status and relationships

	Leeds		National	
	Male	Female	Male	Female
	Mean ± SD (%)	Mean ± SD (%)	%	%
Education				
Achieving a good level of development in the Early Years Foundation Stage Profile [EYFSP] *	45.6 ± 13.4	60.2 ± 13.0	52.0	69.0
Achieve level 2+ key stage 1 reading *	80.1 ± 6.35	88.0 ± 6.87	86.0	92.0
Achieve level 2+ key stage 1 writing *	75.1 ± 7.31	85.5 ± 7.30	80.0	90.0
Pupils at the End of KS4 Achieving 5+ A*-C Including English and Mathematics *	52.4 ± 15.9	62.9 ± 15.2	55.7	65.7
Adults (16–64) with no qualifications	16.1 ± 8.38	16.9 ± 8.82		
Housing				
Aged 16+ living alone	17.2 ± 6.30	17.6 ± 3.76		
Living in council-owned high-rise flats*	62.4 ± 10.1	37.6 ± 10.1		
Living in social housing (aged 16+)	17.7 ± 14.5	19.9 ± 15.2		
Employment				
Unemployed (aged 16+ exc FT students) *	5.80 ± 3.12	3.30 ± 1.92	4.9	3.1
Long term unemployed (aged 16–64) *	2.70 ± 1.66	1.69 ± 1.05	2.2	1.7
Never worked (aged 25+) *	2.48 ± 1.96	6.23 ± 5.94	2.1	5.7
Employees and self-employed (aged 16+ years) working long hours - over 49 h *	9.87 ± 3.58	3.14 ± 1.46	8.5	2.2
claiming Job Seekers Allowance *	4.19 ± 3.51	2.18 ± 1.92	2.7	1.6
Economically inactive due to long-term disability or illness (aged 16+)	4.18 ± 2.32	3.69 ± 1.89	4.1	3.7
Employment and Support Allowance (ESA)	5.88 ± 3.30	5.01 ± 2.67	5.4	4.6
Attendance allowance (aged 65+ years) *	10.5 ± 3.65	16.8 ± 5.86	10.8	18.0
Marital status and relationships				
Single (aged 16–64 years) *	48.4 ± 13.5	42.8 ± 14.7	45.4	38.5
Single (aged 30–49 years) *	39.1 ± 11.3	27.0 ± 7.82	35.4	27.6
Lone parents (aged 16–74) *	0.85 ± 0.43	8.16 ± 4.32	0.8	7.2
Divorced (aged 30–59 years) *	10.5 ± 2.28	13.7 ± 2.70	8.4	11.9

* *P* < 0.01

Boys (aged 5–7 years) were also significantly less likely to achieve level 2+ key stage 1 reading (boys 80.1% ± 6.35%, girls 88.0% ± 6.87%, *P* = 0.00) and writing (boys 75.1% ± 7.31%, girls 85.5% ± 7.30%, *P* = 0.00) (Table 1). In 6 MSOAs in Leeds less than 60% of boys achieved level 2+ in writing, and in 11 MSOAs less than 70% of boys achieved level 2+ reading, with 1 MSOA having only 58% of their boys achieve this level. For girls, all MSOAs had more than 60% of girls reaching this level for writing, with only 6 of the 107 MSOAs in Leeds having less than 70% of girls reach this level for reading.

In 2013, the proportion of boys in Leeds achieving five or more higher grade GCSE passes including English and Maths was significantly lower compared to girls (52.4% ± 15.9% vs. 62.9% ± 15.2%, *P* = 0.00) and was lower compared to boys nationally (55.7%) (Table 1). In 9 MSOAs across Leeds less than 30% of boys are achieving this standard, with all MSOAs seeing more than 30% of girls getting those grades.

Approximately 69% of Looked After Children (LAC) accessing alternative education provision were boys, and the proportion of male LAC achieving a good level of development in the early Years Foundation Stage profile (23%) was similar to that observed in the lowest five ranked MSOAs in Leeds (19–26%). The proportion of male LAC achieving five or more A-C grades at GCSE including English and maths (14%) was less than observed in the lowest ranked MSOA in Leeds (21% for boys).

Across Leeds, 16.1% ± 8.38% of males (16–64) had no qualifications in 2011, however the top ten ranked MSOAs with the highest proportion ranged from 30% to 37% of males with no qualifications.

### Housing

Approximately 17% of men aged 16+ lived alone in Leeds in 2011, which was similar to the number of women (Table 1). In the top ten ranked MSOAs with the highest proportion this was as high as 26.2% to 42.0% of men. Across Leeds as a whole approximately 16% of men in Leeds aged 16+ lived in social housing (rented from the local council or a not-for-profit housing association approved and regulated by Government) in 2011, however in the top ten ranked MSOAs this was as high as 39.4% to 59.1%.

**Fig. 1:**
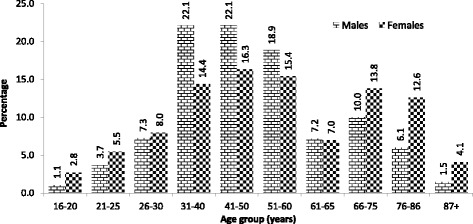
Percentage of men and women living in council owned high-rise flats in Leeds by age group (from Seims & White ^5^)

In April 2015 there were blocks of council-owned high-rise flats in Leeds where 75–85% of residents were males and overall, there was a significantly higher proportion of male residents in this type of housing (62.4% ± 10.1% vs. 37.6% ± 10.1%, *P* = 0.00). The highest proportion of residents within these complexes were aged between 31 and 60 years of age (Fig. 1).

The 2011 census data for Leeds showed that 127 men (aged 16+) were living in a privately owned hostel or temporary shelter for the homeless which is almost double the number of females (64).

### Employment

The number of unemployed men in Leeds, in 2011 was 68% higher compared to females, a gender difference much higher than for England & Wales, where 48% more men were unemployed compared to women. Unemployment was significantly greater among men than women (5.80% ± 3.12% vs. 3.30% ± 1.92%, *P* = 0.00) (Table 1), and the top ten ranked MSOAs with the highest proportion ranged from 10.9%–15.7%.

In 2011, significantly more men were classed as long-term unemployed than women (2.70% ± 1.66% vs. 1.69% ± 1.05%, *P* = 0.00), which was greater than observed for England & Wales (2.2%) (Table 1). The gender gap was also greater in Leeds compared to the data for England & Wales with 60% more men in Leeds classed as long-term unemployed compared to women verses a gap of 32% nationally. The top ten ranked MSOAs with the highest proportion of men (16–64 years) who were long-term unemployed ranged from 5.1% to 7.5%.

In 2011, 2.48% ± 1.96% % of men in Leeds aged 25+ had never worked which was higher than men in England & Wales (2.1%) (Table 1). In the ten highest ranked MSOAs this was as high as 5.2% to 8.2%.

**Fig. 2:**
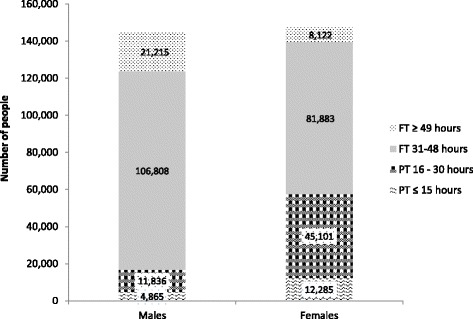
Hours worked by male and female employees (from Seims & White^5^)

**Fig. 3:**
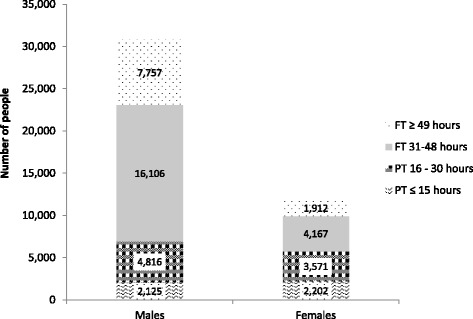
Hours worked by self-employed males and females (from Seims & White^5^)

In 2011, those men in work (employed and self-employed) were significantly more likely to be working full time, with nearly 9.87% ± 3.58% of men in Leeds over the age of 16 years working over 49 h a week compared to 3.14% ± 1.46% of women (*P* = 0.00, (Fig. 2 and Fig. 3). The top ten ranked MSOAs with the highest proportion of male employees (aged 16+ years) working long hours (over 49 h a week) ranged from 10.4% to 13.3% of men.

There were 4.19% ± 3.51% of men claiming Job Seekers Allowance in Leeds in 2014, as compared to 2.18% ± 1.92% of women (*P* = 0.00), but in 3 MSOAs over 14% of the male population were claimants.

In 2011, 4.18% ± 2.32% of men (3.69% ± 1.89% of women) (aged 16+) were economically inactive due to long-term disability or illness (*P* = 0.23), however the top ten ranked MSOAs with the highest proportion ranged from 7.4% to 10.1%. In 2014, 5.88% ± 3.30% of men (aged 16–64) in Leeds claimed Employment and Support Allowance (ESA) as compared to 5.01% ± 2.67% of women (*P* = 0.09), with the top ten ranked MSOAs with the highest proportion of male claimants ranging from 10.9 to 13.0%. The most prominent underlying condition was mental and behavioural disorders, accounting for almost 50% of males claiming ESA. In 2014, 10.5% ± 3.65% of men aged over 65 claimed attendance allowance in Leeds (16.8% ± 5.86% of women, *P* = 0.00), however in the top ten ranked MSOAs this ranged from 14.7 to 25.8% of men in those areas.

### Marital status and relationships

In 2011, 48.4% ± 13.5% of men aged 16–64 years were single, which was significantly higher than for women (42.8% ± 14.7%, *P* = 0.00) (Table 1). Significantly more men within the middle-aged (30–49 year) population of Leeds, were also single men compared to women (39.1% ± 11.3% vs. 27.0% ± 7.82%, *P* = 0.00).

The data from the 2011 Census for Leeds shows that divorced men are typically aged 30–59 years and that approximately 11% of this age group are divorced, however in the ten MSOAs ranked with the highest proportion of divorced men this ranged from 13.2% to 15.9%.

In 2011 there were 2254 lone fathers (aged 16–74) with dependent children in Leeds (0.85% ± 0.43% of men aged 16–74 in Leeds). This was proportionally similar to data for England and Wales (0.8% and 7.2% of the male and female 16–74 year population were lone fathers and mothers respectively).

## Discussion

### Main findings from this study

This is the first study to explore the social determinants of health from a male perspective across an entire city. Our study suggests that there is a clustering of known social determinants that are detrimental to health around deprived areas of Leeds and that there are marked gender differences evident. Although the current analysis did not allow for individual’s experiences to the mapped, it is notable that there was a common group of MSOAs which saw the highest proportion of men struggling with the social determinants of health. Those areas of Leeds that showed poor educational attainment also had a high proportion of long-term unemployed men, a higher proportion of men claiming benefits, more problematic housing and divorced men.

### Comparison with the literature

There are well-established links between poverty and health [52–56], with the Marmot report highlighting that men generally have higher rates of premature death when experiencing social and economic hardship [1]. The health implications for men and boys with regard to worsening socio-economic hardship has also been noted both nationally [57] and internationally [2, 3, 58].

### Implications of the findings

With the move of Public Health into local government, the link between targeting the social determinants of health as a means of improving the health of the local population has been acknowledged, but fiscal constraints mean more focused provision may prove more cost effective.

Planning services for effective public health requires targeting of resources to achieve maximal effect. What has become apparent through the study is the impact of the social determinants on men have been generally under-reported in the literature and this has perhaps left a cohort of vulnerable men hidden from Commissioners’ eyes. The clustering of factors that influence men and their health suggests they need to be recognised and tackled as a whole; focusing on single items negates the complexity of the broader picture.

Breaking the data down by MSOA gives a more detailed view of where services should be targeted and also gives visibility to the social problems that men are facing and a possible explanation for some of the health challenges they face. This is most starkly seen with regard to the high level of suicide in men within Leeds, with over 5 times more men dying as a result of taking their own life as compared to women, with living in the high rise flats, creating the most notable risky setting [13, 59, 60].

The analysis of educational attainment across the City showed that there is a need to consider not just how to get young boys better engaged with schooling, but that support may be needed for men throughout their lifespan. With some areas of Leeds having nearly a third of their male population with no educational qualifications this adds greatly to their risk of unhealthy lifestyles and risk of premature death [55, 61–63]. By targeting boys early in their school life they can be helped to overcome some of their deficits and this has long term benefits with regard to their cognitive skills and achievement, behaviour, mental health, other school related outcomes, and adult outcomes [64].

In support for the need for sex-specific data on the social determinants [32, 36] a key observation made in the Leeds study [13] was that although there were comprehensive locality maps of deprivation produced by the Leeds public health observatory, they rarely offered sex-specific data, leaving the possibility that those most at risk (either male or female) to be missed within any subsequent planning decisions. By making explicit the data on men and women it can also help inform health policies relating to the potential impact of the social world they live in.

The Leeds Report was initiated by the Health and Wellbeing Board and the Director for Public Health, and is now being considered by the Scrutiny Board of the Council, with view to stimulating a City-wide response to the report’s findings. This will be the first time that a whole city has instigated a review of services for men and offers up a model for how other cities can tackle men’s health.

There has already been some response, with, for instance, a new service being introduced into the high rise flats in Leeds. The introduction of the bedroom tax and the system of housing allocation has impacted on where young single unemployed men are being housed, with more now being located in the high rise blocks of flats than previously. The Commissioners have noted that this is creating a new problem, where these mostly white young men are suffering from low self-esteem, depression and social isolation. By offering support on their doorsteps has started to create a safer environment for them and an opportunity to identify physical and emotional problems earlier than previously.

Further research is needed with those living in the high risk areas to determine if they are affected by multiples of the factors identified in this paper, in the same way that are now being identified through the clustering of lifestyle factors such as smoking, drinking and sedentary behaviour [65]. This might enable a much more nuanced level of care planning for those individuals who are the most vulnerable.

### Limitations of this study

The data that has been presented gives an overview of some of the key aspects of the social determinants that could be seen to affect the health of men, however the study was based on available data and was part of a bigger study, and therefore should not be seen as comprehensive.

This is not a longitudinal study and therefore the current data cannot be directly matched. There is a growing realisation of the clustering effect of factors that impact on health and wellbeing [66], however the health data available through the public health observatory does not allow for grouping of social factors at the individual level.

There may well be other levels of difference based on the intersectional factors, such as age, ethnicity, sexuality, and disability; this would need to be incorporated into future studies.

## Conclusion

Although data collected across the city is disaggregated by sex there appears to be a lack of a gendered analysis of its implications for both men and for women. There is great variance in the social worlds of men across a city, which has implications for their health and wellbeing. In areas of deprivation a complex picture of multiple social problems emerges, with marked gender differences in the social determinants of health, with males seeming to be more negatively affected. By targeting men and boys more effectively greater efficiency in service delivery could be obtained. This examination of men’s social circumstances within a city could act as a model for undertaking similar studies both in the UK and elsewhere.

## Abbreviations

ESA: Employment and support allowance
EYFSP: Early years foundation stage profile
GCSE: General Certificate of Secondary Education
LAC: Looked after Children
MSOA: Middle super output area
ONS: Office for National Statistics
